# Management of Sjögren's Syndrome: Present Issues and Future Perspectives

**DOI:** 10.3389/fmed.2021.676885

**Published:** 2021-06-07

**Authors:** Claudio Vitali, Antonina Minniti, Francesca Pignataro, Wanda Maglione, Nicoletta Del Papa

**Affiliations:** ^1^Rheumatology Outpatient Clinics, “Mater Domini” Humanitas Hospital, Castellanza, Italy; ^2^Department of Rheumatology, ASST G. Pini-CTO, Milan, Italy

**Keywords:** Sjögren's syndrome, classification criteria, outcome measures, autoantibodies, biomarkers

## Abstract

In view of the new possibilities for the treatment of primary Sjögren's syndrome (pSS) given by the availability of new biotechnological agents targeting the various molecular and cellular actors of the pathological process of the disease, classification criteria aimed at selecting patients to be enrolled in therapeutic trials, and validated outcome measures to be used as response criteria to these new therapies, have been developed and validated in the last decades. Unfortunately, the therapeutic trials so far completed with these new treatments have yielded unsatisfactory or only partially positive results. The main issues that have been evoked to justify the poor results of the new therapeutic attempts are: (i) the extreme variability of the disease phenotypes of the patients enrolled in the trials, which are dependent on different underlying patterns of biological mechanisms, (ii) the fact that the disease has a long indolent course, and that most of the enrolled patients might already have irreversible clinical features. The advances in the research of new disease biomarkers that can better distinguish the different clinical phenotypes of patients and diagnose the disease in an earlier phase are also discussed.

## Introduction

Primary Sjögren's syndrome (pSS) is a systemic autoimmune disorder whose characteristic pathologic feature is the lymphocytic infiltration of exocrine glands, namely the salivary and lachrymal glands, with a slow progressive loss of function and, as a consequence of that, oral and ocular dryness (1). Middle-aged women are predominantly affected by pSS, whilst the disease is more rarely observed in men (female/male ratio 9:1). The real prevalence of the disease in the general population has not been precisely defined, being reported from 0.1 to 3 per 1,000 in different epidemiologic surveys (2–4).

The clinical spectrum of pSS is extremely variable. In around 50% of the patients the clinical symptoms related to glandular involvement (GI) are accompanied by extraglandular manifestations (EGMs) that mainly involve joints, kidney, lung, peripheral nervous system, and small vessels (5). Severe fatigue and widespread pain (WP) are other characteristic features of the disorder (6), often associate with a depressive state. Finally, it has been shown that, in a limited number of cases, the strong B-cell polyclonal proliferation that characterizes the infiltration of target tissues (namely the salivary glands), may evolve to a selective monoclonal proliferation and later to the development of lymphoid malignancies (7, 8). As well as in its primary form, SS has also been reported in association with systemic sclerosis, systemic lupus, and rheumatoid arthritis. In these cases SS can be defined

as “associated” with other systemic autoimmune diseases. The clinical, serological and pathological features of these associated forms may differ slightly or greatly from those observed in pSS (9). Thus, the data presented and discussed in this paper are only referred to the primary variant of the syndrome.

## Summary on the Pathogenesis of PSS

**Figure 1:**
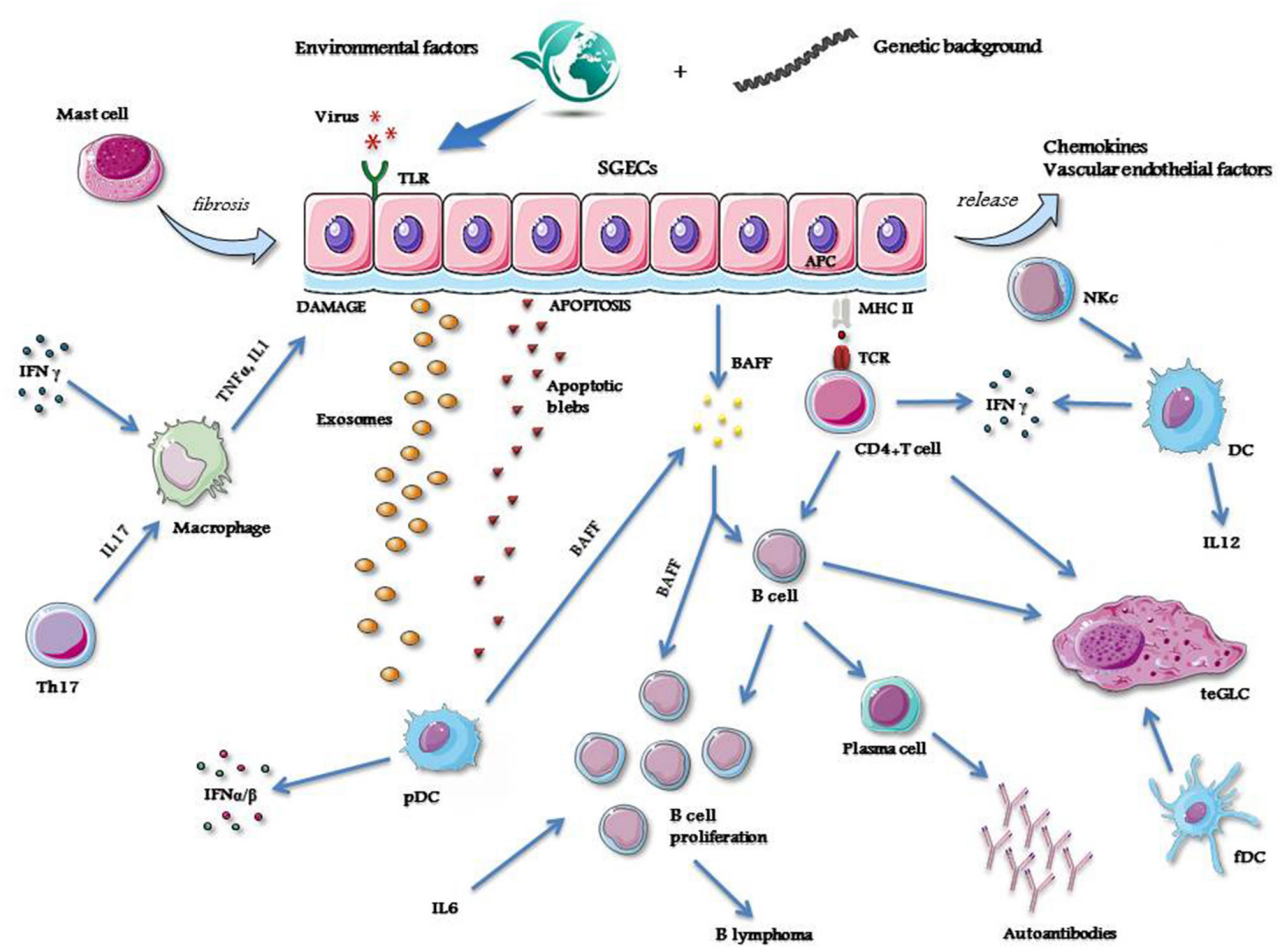
A simplified view showing the mechanisms, cells and molecules operating as the main actors of the pathological process of pSS. APC, antigen presenting cell; BAFF, B cell activating factor; DC, dendritic cell; fDC, follicular dendritic cell; IFN, interferon; IL, interleukin; MHC, major histocompatibility complex molecule; NKc, natural killer cell; pDC, plasmacytoid dendritic cell; SGECs, salivary glands epithelial cells; TCR, T cell receptor; teGLC, tertiary ectopic germinal-like center; Th, T helper cell; TLR, Toll like receptor; TNFα, tumor necrosis factor alfa. Credit: modified from Smart Servier Medical Art, https://smart.servier.com.

A simplified view showing the different mechanisms, cells and molecules operating as the main actors of the pathological process of pSS is represented in Figure 1.

As in other autoimmune diseases, also in pSS the development of the pathological process requires the intervention of different factors. An incident viral infection may trigger the disorder (10, 11), and different viruses such as hepatitis C virus (HCV), human immunodeficiency virus (HIV), Epstein–Barr virus (EBV), cytomegalovirus (CMV), coxsackievirus and human T-lymphotropic virus-1 (HTLV-1) have been suggested as possible inducers of the disease, starting from some observations demonstrating that these viruses may cause persistent infection of the salivary glands and lead to organ damage, thus causing dry mouth (10, 11). However, the pathological features of the sialoadenitis caused by these viral infections are consistently different (12).

Genome wide association studies (GWAS) have identified alleles belonging to class II major histocompatibility complex (MHC), namely those of the HLA-DR and HLA-DQ isotypes, which are closely associated with pSS. In addition, significant association with the disease has also been reported for a number of non-MHC genes, namely for genes belonging to the pathway of interferon (IFN) signaling (13, 14). Thus, it has been postulated that these susceptibility genes may play important roles in the activation of some crucial pathogenetic processes of the disease (15). However, most of these upregulated genes, including those involved in IFN pathway activation, are not specific for pSS but are shared by most members of the systemic autoimmune disorders (16).

In genetically susceptible subjects, environmental stimuli may trigger salivary gland epithelial cells (SGECs) through specific Toll-like receptor (TLR) activation (17). SGECs are not innocent bystanders or the ordinary victims of the inflammatory cascade, but these cells, once activated, orchestrate the whole pathologic process of the disease (18) by inducing the production of a number of chemokines and vascular endothelial factors which strongly contribute to attract immune and inflammatory cells, like natural killer (NK) cells, T-cells, B-cells and macrophages (19). All of these cell types variably contribute to the formation of inflammatory infiltrates that, in some cases, may assume the aspect of tertiary ectopic germinal-like centers (teGLCs) (20). The attracted macrophages produce large amounts of inflammatory cytokines, namely IL-1 and TNFα, which lead to local tissue damage (21, 22). In addition, in activated SGECs the apoptotic mechanism is triggered (23), with the subsequent release of autoantigens into the environment via autoantigen-containing apoptotic blebs and exosomes (24, 25). SGECs also have the ability to act as non-professional antigen-presenting cells, as demonstrated by the expression of class I and II MHC molecules on their surface (26). Thus, these cells may present autoantigens to immune-competent cells such as CD4+T-cells (27). These T-cells, by the subsequent interaction with B-cells (28), can drive the autoantibody production via the B-cell lineage terminals, the plasma cells (29).

The autoantigen materials released by SGECs activate plasmacytoid dendritic cells (pDC) (30). These cells are able to produce type I IFNs (31, 32), and, together with SGECs, B cell activating factor (BAFF) (33). The latter cytokine, along with IL-6, plays a fundamental role in B cell proliferation and survival, and, in the later phase of the disease, may induce lymphomagenesis (34). Type I IFNs act by transcription of IFN-related genes which contribute to the autocrine and paracrine maintenance of the inflammatory state (35).

NK cells induce the activation of dendritic cells (DC) which are the main producers, together with T helper (h) cells, of other important inflammatory cytokines, such as IFNγ and IL-12 (36–38). IFNγ and IL-17 - the latter produced by Th-17 cells, a subtype of Th cells—contribute to maintain the activation of macrophages and then to the related production of inflammatory cytokines (39). Finally, follicular (f) DCs actively participate in the organization of the inflammatory infiltrates in the glandular tissue, driving the formation of teGLCs (40). Mast cells also take also part in the pathological process in the salivary glands by inducing local fibrotic changes (41).

## Classification Criteria and Outcome Measures

During the past 20 years, different criteria have been proposed for the classification of pSS. The most widely used criteria set has been the American European Consensus Group (AECG) criteria (42). In 2012, new classification criteria were proposed by the American College of Rheumatology (ACR) (43). A synthesis of previous classification criteria was obtained by a collaborative effort of the ACR and European League Against Rheumatism (EULAR) (44, 45). This set of criteria utilizes a weighted sum of five selected diagnostic items to allow classifying a patient as having pSS (Table 1). In this set the highest weight (3 points) is attributed to both anti-SSA/Ro antibody positivity and presence of a focus score of at least 1 in minor salivary gland biopsy (MSGB), while only 1 point is given to each of the other three items. The focus is defined as an agglomerate of at least 50 mononuclear cells in salivary tissue, and the focus score is calculated as the number of foci observed in 4 sq mm of tissue. Since applying the ACR-EULAR criteria a minimal score of 4 is needed to classify a patient as having pSS, it appears evident that, as in AECG criteria, the presence of either anti-SSA/Ro antibodies or focus score ≥ 1 is mandatory for the classification (42, 44, 45).

**Table 1:** The 2016 ACR/EULAR classification criteria for pSS.

**Item**	**Weight/score**	**Rules for classification**
Labial salivary gland with focal lymphocytic sialadenitis and focus score of ≥1 foci/4 sq mm	3	To be applied to any individual who meets the inclusion criteria (presence of ocular and/or oral dryness) with at least one symptom of ocular or oral dryness or ESSDAI ≥ 1.
Presence of anti-SSA/Ro-antibodies	3	
Ocular Staining Score (OSS) ≥ 5 (or van Bijsterveld score ≥ 4)	1	Absence of any of the conditions listed below as exclusion criteria.
Schirmer's test ≤ 5 mm/5 min in at least one eye	1	A score of ≥ 4 when the weights from the five criteria items are summed.
Unstimulated whole saliva flow rate ≤ 0.1 mL/min	1	

*Exclusion criteria: history of head and neck radiation treatment, active hepatitis C infection (with confirmation by PCR), Acquired Immune Deficiency Syndrome (AIDS), sarcoidosis, amyloidosis, graft-versus-host disease, and IgG4-related disease*.

As in other systemic autoimmune diseases, the need to assess the different levels of disease activity and damage, namely when old and new therapies are tested in clinical trials, have induced the scientific community to define and validate outcome instruments for both these disease status entities also in pSS. A joint effort of the EULAR SS Task Force has produced two outcome measures for the evaluation of the different levels of disease activity: the EULAR SS Patient-Reported Index (ESSPRI) for patient symptoms, as dryness, pain and fatigue (46), and the EULAR SS Disease Activity Index (ESSDAI) for systemic features (47). Thus, the availability of validated outcome measures aimed at assessing different domains of pSS, has made it easier to apply an evidence-based methodology in performing therapeutic trials for this disorder (48). Furthermore, two indices derived from two national studies carried out in Italy and England have been proposed to evaluate the accumulated damage caused by disease progression (49, 50).

Other instruments have been proposed and validated for specific features of pSS, such as Functional Assessment of Chronic Illness Therapy (FACIT) (51) and Profile of Fatigue (PROF) for fatigue (52), Sicca Symptoms Inventory (SSI) (53) for sicca complaints, Hospital Anxiety and Depression Scale (HADS), for the often-associated affective disorders (54). An integrated short form (SF) questionnaire where fatigue, and (A) pain discomfort (D) together with sicca complaints are simultaneously investigated (PROFAD-SSI-SF), has been also proposed and applied in some surveys in pSS patients (55).

Obviously, further efforts are needed to improve the reliability of the outcome measures to be adopted in future therapeutic trials. Composite instruments that can separately assess the different domains present in the disease spectrum are under investigation (56).

## Ocular and Salivary Assessment

The commonly used ophthalmological tests for the assessment of lachrymal production and function are the Schirmer's test and break up time (BUT), while dye tests are used to recognize and quantify damages in the dry conjunctival and corneal surface (57).

Salivary dysfunction is usually measured by collecting the whole saliva volume produced in a given time with or without stimulation (58). Salivary glands function can also be precisely explored by dynamic salivary scintigraphy (59).

Salivary gland ultrasound (SGUS) examination is now the most common method used to evaluate the anatomical changes related to pSS in this target organ. Although largely used in clinical practice, international agreement on how to perform this imaging technique and evaluate the abnormalities observed in major salivary glands is still lacking (60). However, the presence of hypoecogenic areas in the glands is considered the most specific finding observed in patients with pSS (61). Despite the potential usefulness of SGUS in diagnosis and classification of pSS, the value of SGUS to assess disease activity and disease progression and to detect salivary gland lymphoma needs to be established. Different SGUS scores have been proposed and some of them seem to correlate with objective salivary gland function, as unstimulated salivary flow rates. Several studies showed associations between SGUS scores and clinical parameters of disease activity, such as ESSDAI scores, IgG levels, and rheumatoid factor levels. In contrast, other studies have suggested that hypoechogenic areas reflect the level of damage of the glands. These discrepancies can be explained by differences in patient characteristics between cohorts [reviewed in van Ginkel et al. (62)].

## Histopathologic Assessment

Although biopsy of major salivary glands has been proposed, it is performed only in few centers, since this procedure is still considered rather invasive and not completely free of complications (63). MSGB, performed in the middle of the lateral part of the inferior lip, is the most commonly used and almost completely safe procedure to obtain salivary tissue to be analyzed for diagnostic and investigative purposes (64). An agreement has been reached by a board of expert on the precise methodology to perform MSGB and analyse the obtained tissue (65). Since little data exist on the natural evolution of the histopathological changes in pSS, the advantages of re-biopsy during therapeutic trials are still the object of debate. Furthermore, ethical concerns were raised about performing repeated biopsies on patients treated with placebo. However, the demonstration of improvement in biopsy scores in pilot studies, even after exclusion of placebo group, could be useful to justify the introduction of MSGB as an additional end point in future studies (65).

From this point of view, particular interest should be reserved to the prospective follow up study conducted in two centers in Italy (66). In this study, two MSGBs were obtained at the time of inclusion and at week 120 in patients treated with conventional disease modifying anti-rheumatic drugs (DMARDs) and in patients treated with rituximab. In contrast to data observed in MSGBs of patients treated with DMARDs, a strong reduction of the focus score and teGLC number was found in second biopsies performed in rituximab-treated patients.

## The Clinical Spectrum of PSS

The pathological mechanisms that are summarized in the previous paragraph are not simultaneously or entirely active in all the patients, but may have a variable expression in subsets of patients showing different clinical phenotypes. It has been shown that this variability is strongly conditioned by gender, race, presence of specific genetic background, and exposure to different environmental factors (67).

The disease can be limited to glandular manifestations that cause symptoms of dryness such as dry mouth, eyes, and also dry skin and dry vagina, as a result of the inflammatory aggression and infiltration of the involved exocrine glands (68). A similar aggression of other extraglandular epithelial structures may cause anatomic damage and dysfunction in various organs such as lung, kidneys and liver (68). Moreover, extraglandular and extra-epithelial immune-complex-mediated deposition may induce a series of systemic manifestations generally due to microvascular inflammatory involvement in the various compartments. These are mainly represented by Raynaud's phenomenon, purpuric cryoglobulinemic vasculitis, glomerulonephritis and peripheral neuropathies (68). Finally, the continuous overstimulation of autoreactive B cell clones may induce, in around 5% of pSS population, a B cell lymphoma, which is usually a low grade indolent lymphoma and in a low percentage a more aggressive large cell lymphoma (7).

The clinical manifestations described as belonging to the clinical spectrum of pSS are listed in Table 2.

**Table 2:** Clinical features of pSS subdivided in glandular, extraglandular epithelial, and extraglandular non-epithelial manifestations.

*General and constitutional symptoms* • Fever • Fatigue • Widespread pain *Uncertain classification* • Small vessel neuropathy • Depressive state • Mild cognitive dysfunction *Glandular manifestations* • Dry eye • Dry mouth • Dry skin • Dry vagina • Dry nose • Dry trachea *Extraglandular epithelial manifestations* Lung: • Small airway disease • Bronchiolitis • Alveolitis inducing interstitial involvement (LIP, UIP, NSIP) Liver: • Autoimmune hepatitis • Autoimmune cholangitis Renal tubuli and bladder: • Tubulo-interstitial nephritis • Renal tubular acidosis (typically type I, less commonly type II) • Renal calcinosis and stones • Interstitial cystitis	*Extraglandular non-epithelial manifestations* Joints: • Arthralgia • Non-erosive arthritis Small vessels: • Raynaud's phenomenon • Urticarial vasculitis • Cryoglobulinemic purpura • Annular erythema/subacute cutaneous lupus erythematosus Haematological manifestations: • Leukopenia (lymphopenia and/or neutropenia) • Thrombocytopenia • Haemolitic anemia Renal glomeruli: • cryoglobulin-mediated membranoproliferative glomerulonephritis • IgA nephropathy Peripheral nervous system manifestations: • Axonal sensory polyneuropathy • Axonal sensorimotor polyneuropathy • Autonomic neuropathy • Cranial neuropathies (II, V, VII, and VIII) • Mononeuritis multiplex • Ganglionopathy Central nervous system manifestations: • Lymphocytic meningitis • Multiple sclerosis-like disease • Transverse myelitis

*General and constitutional symptoms and some features of uncertain classification are separately classified*.

The composition of inflammatory infiltrates, the presence of teGLCs, activation in the target tissue and in peripheral blood mononuclear cells (PBMCs) of specific biological pathways such as type I and type II IFN signature, and the amount of inflammatory cytokines, can be significantly different in subsets of patients characterized, at one extreme by a disease limited to GI or, at the other, complicated by the systemic EGMs (69, 70).

**Figure 2:**
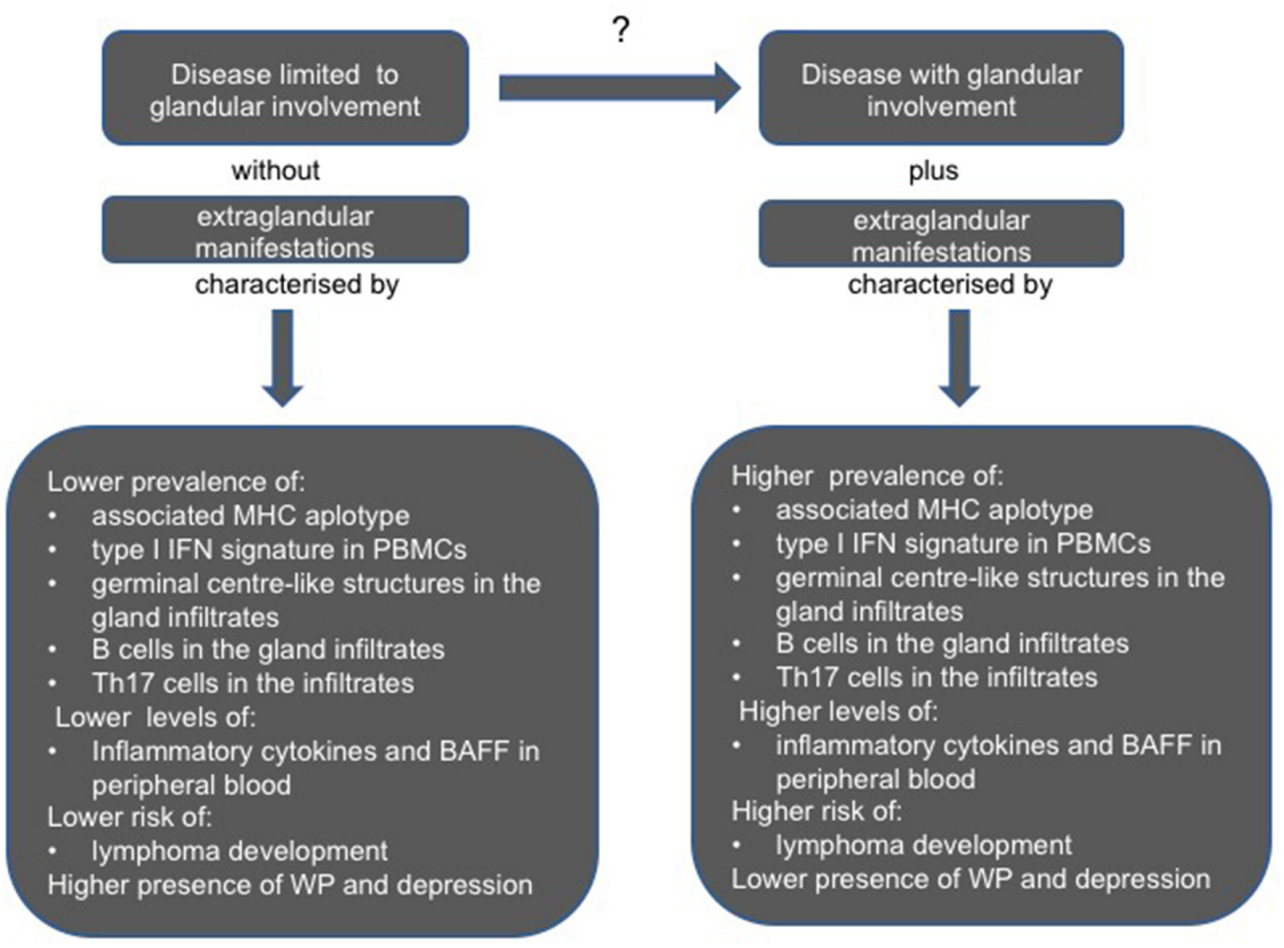
Clinical, serological, and pathological differences between pSS patients with a disease limited to glandular involvement and absence of extraglandular involvement, and those with glandular features plus extraglandular manifestations. BAFF, B cell activating factor; IFN, interferon; MHC, major histocompatibility complex molecule; PBMCs, peripheral blood mononuclear cells; Th, T helper cell; WP, widespread pain.

Figure 2 summarizes the biological and pathological differences described in these different subsets of pSS patients.

## Clinical and Biological Markers Presently Useful to Characterize Patients with Pss

In the wide spectrum of pSS a number clinical, serologic, and histologic features have been described as predictors of disease severity, presence of systemic manifestations and lymphoma development. Recurrent salivary gland enlargement, palpable purpura due to cryoglobulinemic vasculitis, cervical lymphadenopathy and splenomegaly have been reported as independent clinical risk factors for the development of lymphoma (71–73). Older age and male gender has also been given as the demographic factors that may predispose to lymphoma development. Finally, it has also been shown that some serologic and hematologic markers have a predictive role of lymphoma development (74). These are the presence of type II cryoglobulins, rheumatoid factor, and low levels of complement C4 fraction in the serum (75, 76), as well as the finding of leukopenia, neutropenia and lymphopenia, particularly CD4 lymphopenia, in the peripheral blood (8, 72, 74, 77). The fact that the presence of teGLCs in MSGBs may be associated with future lymphoma development has also been reported (78, 79), but this finding has not been confirmed in other studies (80).

High focus score, anti-SSA/Ro and SSB/La, antibodies, younger age at disease onset have been indicated as findings linked to a more severe systemic disease (81, 82), whilst patients without antibodies and a low degree of focus score often have a disease limited to GI, lower levels of inflammatory cytokines in peripheral blood and may complain more frequently of severe fatigue, depressive state and WP (83).

Pregnant women with anti-SSA/Ro and SSB/La run a certain risk of having a fetus that develops heart rhythm abnormalities, and namely complete heart block (84).

In some patients the serological detection of anti-centromere antibodies (ACA) and anti-cyclic citrullinated proteins antibodies (anti-CCP) may indicate the development of clinical features overlapping with systemic sclerosis in its limited cutaneous variant (lcSSc), and with rheumatoid-like erosive arthritis, respectively (85, 86). Finally, it has been reported that the serological finding of anti-U1-ribonuclear proteins (anti-U1-RNP) antibodies is associated with pulmonary involvement (87).

## Therapeutic Approach

EULAR therapeutic recommendations for pSS have recently published (88). These recommendations are based on the few recent studies in which some evidence of efficacy for the management of patients with pSS has been found. However, for the majority of therapeutic issues, the recommendations are based on expert opinion, and then derived from discussions among a large international task force (88). Until their eventual updating in the next years, these recommendations represent the state of art of the therapeutic approach to patients with pSS, and should be carefully considered as useful guidelines to take into account for clinicians when managing patients with the disorder. In addition to topical treatment of dry eye and dry mouth that requires the expertise of ophthalmologists and stomatologists, the general treatment is usually entrusted to rheumatologists.

The therapeutic management of pSS has not changed substantially in recent decades (89) and is still based on the symptomatic treatment of sicca symptomatology and a variety of immunosuppressive agents for systemic features.

Briefly, some benefit of muscarinic receptor agonists (pilocarpine and cevimeline) for the relief of oral dryness and ocular dryness symptoms has been demonstrated in randomized clinical trials (RCTs) (90). Cyclosporine ophthalmic emulsion are approved for dry eye and are widely used by ophthalmologists, namely when the simple application of artificial tears in partially ineffective (91, 92). Studies of systemic conventional immunosuppressive drugs (such as prednisone, cyclosporine A, azathioprine, methotrexate) and other interventions such as dehydroepiandrosterone, nizatidine, and rebamipide are generally considered to be ineffective in controlling sicca symptoms, although modest benefits have been reported for some drugs (90, 93–96). Synthetic or biologic disease modifying therapies which have been tested and approved for the treatment of many other autoimmune diseases, have failed to demonstrate significant clinical effects in pSS. Thus, their use in this disease remains empirical or limited to some subsets of patients as suggested by some studies carried out on a limited number of patients and by *post-hoc* analyses performed in the few completed RCTs (88).

## The Use of Innovative Target Therapies and Reasons for the Failure of Rcts in PSS

Advances in the knowledge of the biological pathways, cell types and molecules that play fundamental roles in the development and progression of pSS, and the contemporary availability of biotechnological target therapies capable of interfering or blocking most of the key points in this pathological cascade, have opened up a large number of new therapeutic possibilities for the management of this disease. However, the recent findings of the RCTs performed in patients with pSS are almost all completely negative. Different reasons can be put forward as possible causes of this negative outcome.

### Insufficient Time Duration of Therapeutic Trials

In most clinical trials, the observation time is limited to 24 or 48 weeks. For pSS, a chronic and slowly progressing disease, this observation period is relatively short, and probably not sufficient to capture a significant improvement in clinical indicators. Therefore, in clinical trials, the application of reliable biomarkers for early diagnosis and patient recruitment, as well as prolonged observation time, will help to demonstrate the potential therapeutic effects of biotherapy in at least certain aspects of the disease (97).

### Insufficient Preliminary Characterization of Patients for Adoption of Target Therapies

Some clinical trials targeting TNFα (by infliximab and etanercept) and IL-1 (by anakinra, an IL-1–receptor antagonist) failed to demonstrate efficacy in pSS (98–100). Similarly, a recent trial in which an anti-IL-6 receptor (tocilizumab) was employed did not demonstrate an improvement of systemic involvement and symptoms over 24 weeks of treatment compared with placebo (101). Another target therapy attempt was carried out with abatacept. This molecular construct interferes with the CD80/CD86-CD28 costimulatory system, leading to the prevention of T-cell activation (102). The results of a recent RCT with abatacept are not consistent (103). In contrast, some previous studies, carried out on more limited numbers of patients with a relatively short disease duration, showed an improvement of disease activity (using the ESSDAI score), and also a reduction in cytokine and autoantibody levels (104, 105).

The efficacy of long-term treatment of SS with belimumab, a monoclonal antibodies targeting the BAFF, in a 1-year open-label trial in 30 patients positive for anti-SSA or anti-SSB antibodies characterized by systemic complications or persistent salivary gland enlargement or early disease or biomarkers of B-cell activation. The improvement in the ESSDAI and ESSPRI scores observed at week 28 showed a trend to further improvement at week 52, and the amelioration of peculiar ESSDAI domains (glandular, lymphadenopathy, articular) appeared of particular relevance (106). A RCT designed to understand the efficacy, safety and tolerability profile of belimumab/rituximab co-administration and of belimumab monotherapy in patients with active pSS is ongoing (ClinicalTrials.gov Identifier: NCT026315).

Generally speaking, in the majority of the trials performed the inclusion criteria were variable and often not sufficiently defined, being limited to the need to meet classification criteria or reach a minimal ESSDAI score as an indicator of a certain degree of systemic disease activity, without a precise distinction between the different domains determining the ESSDAI increase, i.e., between manifestations probably due inflammatory involvement of extraglandular epithelial tissues, and features more likely ascribed to B cell hyperactivity and immune-complex deposition in skin, renal, and peripheral nervous system small vessels. This may have greatly influenced the results considering that any tested agent could act positively or, on the contrary be ineffective in the pathological mechanisms underlying the different clinical features present in non-homogeneous subgroups of patients.

Since the presence of autoantibodies, hyper-gammaglobulinemia and increased risk of B cell lymphoma highlighted the importance of B-cells in the pathogenesis of pSS, rituximab has been one of the most investigated target therapies in this disease. Rituximab is a chimeric monoclonal antibody that targets the CD20 molecule expressed by most B-cells, leading to apoptosis or cellular cytotoxicity and then to B-cell depletion (107, 108). On the whole, controlled trials using rituximab failed to show significant clinical efficacy in the enrolled cohorts of patients (109, 110). Devauchelle-Pensec et al. (109) reported the results of a conducted in 120 patients with pSS, characterized by the presence of scores of 50 mm or greater on at least 2 of 4 visual analog scales (VASs) (global disease, pain, fatigue, and dryness) and recent-onset (<10 years). The patients were randomized (1:1 ratio) to receive rituximab (1 g at weeks 0 and 2) or placebo. Primary end point was improvement of at least 30 mm in 2 of 4 VASs by week 24. No significant difference between groups in the primary end point was found, although the proportion of patients with at least 30-mm decreases in at least two of the four VAS scores was higher in the rituximab group at week 6. A possible explanation for these negative results could be the low disease activity at baseline and the primary end point that may have been insensitive to detect clinically changes.

Bowman et al. (110) published the results of an additional RCT with rituximab conducted in 133 anti-SSA/Ro positive patients with primary SS, symptomatic fatigue, and oral dryness. Patients were randomized to receive either rituximab or placebo. The primary end point was the proportion of patients achieving a 30% reduction in either fatigue or oral dryness at 48 weeks, as measured by VAS. Other outcome measures included salivary and lacrimal flow rates, quality of life, ESSPRI and ESSDAI scores. In rituximab-treated patients, with respect to placebo group, there were no significant improvements in any outcome measure except for unstimulated salivary flow. A possible explanation of the negative results of this study could be that the chosen end points were not closely related to B cell activity.

Some open studies and retrospective surveys have shown that rituximab can be effective in at least one of the systemic outcomes analyzed (organ-specific response, ESSDAI reduction, biological markers and/or glucocorticoid reduction), and, as expected, appeared particularly effective in some biological markers of B cell hyperactivity (66, 111, 112).

By and large, these results allow validating the use of rituximab in the management of selected subsets of patients of pSS with specific clinical manifestations (88). This recommendation implies the explicit acceptance by the scientific community of the fact that the illusion of threating all patients with pSS with the same therapy should be abandoned. A new path has to be taken, that of better biological and pathological characterizations of different phenotypes of patients with the purpose of differentiating the therapeutic approach in each subgroup of them.

Other target therapies have been tested in trials in which a limited number of patients were enrolled, or are under investigation. These new therapeutic agents are aimed at modulating the action of different molecules or pathways which are considered essential in the pathogenesis of pSS, such as for instance TLRs, IFNs and other key cytokines, chemokines, and JAK-STAT signal transducer systems. The results of these RCTs will be available in the near future (113).

### Failure of Enrolling Patients in the Early Reversible Phase of the Disease

The classification criteria for pSS are based on the typical dryness signs and symptoms of the disease, serological evidence of specific autoantibodies, and histopathological evidence of focal lymphocytic sialoadenitis (44, 45). Since pSS is a slowly progressive often indolent disease, classification criteria which are commonly used to collect patients to be enrolled in therapeutic trials, might be unable to capture patients in the early stage of the disease, in whom a therapeutic intervention may have greater chances of modifying the natural history and the final outcome of the patients. These patients with an early phase of the disease may progress and meet the classification criteria only after a certain number of years when the pathological changes have become chronic and the functional organ derangement could be irreversible. This is one of the issues often raised to justify the failure of different therapies in the disease. In planning trials, some rules have been adopted to reduce this risk, such as the inclusion of patients with a shorter disease duration and with evidence of residual glandular function (104). However, it is obvious that the inclusion of patients in the early stages may increase the probability of success of any therapeutic approach. Early patients could be those with pSS-related autoantibodies but lacking clinical symptoms, or those with clinical dryness symptoms but lacking serological or histopathological evidence.

It has been demonstrated that antibodies are present many years before the clinical onset of pSS, and the number of autoantibodies increases during disease progression (114, 115). On the other hand, some patients may have dryness symptoms in the early stages, but serological or histopathological evidence of pSS are lacking in this disease phase. A prospective study carried out on patients with sicca symptoms showed that some of these patients progressed to clinically evident pSS after several years, and in a percentage of them anti-SSA/Ro and anti-SSB/La antibodies appeared during the observation time, together with an increased degree of lymphocytic infiltrates in MSGBs (116).

## Need for New Biological Markers

There is increasing agreement among the experts that there is a need for new biomarkers which may allow diagnosing the disease in an early phase and better distinguishing its different phenotypes (117).

Some more recently described autoantibodies, like those against aquaporin 5 (AQ5-Ab), a water permeable channel located in the epithelial cells of salivary glands, and anti-carbonic anhydrase I seem to be associated with specific clinical and serological features (118, 119). Elevated levels in the serum of some cytokines have been reported to be associated with the formation of teGLCs (CXCL12) (120), B cell hyperactivity (CXCL13) (121), while high expression of CXCL13 and CCL21 in MSGBs has been reported to be related to more severe lymphoid proliferation (122, 123) and together with that of CXCL12 to the presence of lymphoma (124). Patients with high BAFF levels have more pronounced B cell activation and a less satisfactory response to anti-CD20 B-cell depleting therapy (125).

Sialochemistry investigations in saliva and tears have performed to identify potential diagnostic biomarkers for pSS (126–129). In these studies, cathepsin S has been identified as a helpful biomarker in identifying patients with pSS, namely those in the early stages (130, 131).

Studies on proteomic profile of saliva or tears through mass spectrometry has been used as another method to identify biomarkers in these secretes which may have a diagnostic potential for pSS (132–134). Although some molecules have been found to be promising diagnostic markers for pSS in different studies, the results of these proteomic analyses are not completely convincing (135).

In recent years, transcriptional analyses have yielded very interesting results (136–138), showing the over expression of type I and II IFN-inducible genes in both PBMCs and in glandular tissue, the so-called type I and type II IFN signature. While type I signature in PBMCs appears to be associated with a higher ESSDAI score, signs of systemic involvement and some specific serological abnormalities (137), the predominance of type II signature in glandular tissue has been reported to be a marker of a possible lymphoma development (139–141).

Microarray analyses of a large set of genes in PBMCs have also demonstrated that the gene profile activation is completely different in patients with systemic features and high ESSDAI score with respect to patients with disease limited to GI and characterized by WP (142).

Epigenetic studies on the regulatory role of microRNAs and long non-coding RNAs have revealed that specific probes are significantly overexpressed in patients with pSS, and that they could assume a diagnostic role in this disorder in the near future (143–145). On the contrary, the level of other microRNAs has been found to be reduced in pSS patients with lymphoma or pre-lymphoma condition (146).

Some novel autoantibodies have rather recently been described in patients with pSS and proposed as new biomarkers for this disease (147, 148). Among these autoantibodies, the diagnostic relevance of anti-salivary protein-1 (Anti-SP1), anti-carbonic anhydrase 6 (Anti-CA6) and anti-parotid secretory protein (Anti-PSP) autoantibodies have been investigated (149, 150), and the fact that these may appear in the early disease stages has been highlighted (151–153). This may be particularly important from the diagnostic point of view in the absence traditional autoantibodies (153). Autoantibodies to muscarinic acetylcholine receptor M3 have been identified in a not relevant proportion of pSS patients (154), and together with anti-AQP5 may have a direct role in causing functional and anatomical damage to SGECs (118, 155). It has been reported that both these autoantibodies can appear in the early stages of pSS, thus making it possible to define in advance the diagnosis of the disease (156, 157).

## Conclusion

pSS has long been an orphan disorder, since no therapy has demonstrated to be really effective. The great progress made in the knowledge on the pathologic aspects and biological mechanisms of the disease, and the entry into the therapeutic armamentarium of the innovative target therapies, has opened up new horizons in the treatment of these patients. However, the unsatisfactory results obtained in large RCTs carried out so far, where the new biological agents were tested, have been really disappointing. However, a thorough reflection of the reasons for such negative data greatly reinforces the belief that the large variability of clinical and biological phenotypes of patients with pSS makes it very unlikely that a single therapy will give positive results in all patients. This implies that a better definition of the pathological and biological profile in the different subgroups of patients is certainly needed in order to choose the most appropriate therapy to be tested and then used in homogeneous subsets of patients (tailored therapy), or even in single patient (personalized medicine). Innovative biological technologies like proteomic, and transcriptomic analyses to be applied in peripheral blood, salivary and lachrymal gland secretes and in target tissues may offer new possibilities for such a purpose (158).

The failure of any tested therapy could be also ascribed to the fact that the chronic indolent course of the disease may induce the clinician to define the diagnosis in late phase of its course, when most of the lesions have probably become irreversible. It is necessary to define new pathological, serological and biological diagnostic markers which may enable the clinician to recognize the disease in an earlier phase, and treat it with an increased possibility of success.

## Author Contributions

All authors listed have made a substantial, direct and intellectual contribution to the work, and approved it for publication.

## Conflict of Interest

The authors declare that the research was conducted in the absence of any commercial or financial relationships that could be construed as a potential conflict of interest.
